# Digital Media Usage Behavior and Its Impact on the Physician-Patient Relationship: Cross-Sectional Study Among Individuals Affected by Psoriasis in Germany

**DOI:** 10.2196/57823

**Published:** 2024-08-07

**Authors:** Mert Ege Erbas, Stefanie Ziehfreund, Hannah Wecker, Tilo Biedermann, Alexander Zink

**Affiliations:** 1 Department of Dermatology and Allergy School of Medicine Technical University of Munich Munich Germany; 2 Division of Dermatology and Venereology Department of Medicine Solna Karolinska Institute Stockholm Sweden

**Keywords:** psoriasis, dermatology, digital health, digital media, internet use, questionnaire, physician-patient relationship

## Abstract

**Background:**

Psoriasis is a chronic skin disorder with a high burden of disease. People affected with psoriasis increasingly use the internet for health-related reasons, especially those with younger age, higher education, and higher disease severity. Despite advantages such as enhancing the individuals’ knowledge with the use of digital media for health-related issues, disadvantages were also present such as quality control, and variability in the individuals’ health information literacy. While patients with psoriasis within medical settings generally trust physicians over digital media, they commonly withhold their web-based research findings from health care providers.

**Objective:**

The study aims to (1) identify further factors associated with regular psoriasis-related internet use, (2) rank specific digital media platforms used, and (3) examine digital media within the physician-patient relationship among individuals with and without dermatological treatment.

**Methods:**

A cross-sectional, questionnaire-based study was conducted among individuals with self-reported psoriasis in Germany between September 2021 and February 2022. Participants were recruited via digital media platforms and in person at a University Hospital Department of Dermatology in southern Germany. The questionnaire asked about demographic and medical information, individual psoriasis-related digital media use, and the impact of digital media on the physician-patient relationship. Data were analyzed descriptively, and logistic regression models were performed to assess the factors associated with regular psoriasis-related internet use.

**Results:**

Among 321 individuals with a median age of 53 (IQR 41-61) years (nonnormally distributed; females: 195/321), female sex, shorter disease duration, moderate mental burden of disease, and good self-assessed psoriasis-related knowledge were associated with regular psoriasis-related internet use. Of the 188 participants with a mean age of 51.2 (SD 13.9) years (normally distributed) who used digital media 106 (56.4%) usually searched for information on psoriasis-based websites and 98 (52.1%) on search engines, primarily for obtaining information about the disease and therapy options, while social media were less frequently used (49/188, 26.1%). Nearly two-thirds of internet users (125/188) claimed that their physicians did not recommend digital media platforms. About 44% (82/188) of the individuals reported to seek for additional information due to the insufficient information provided by their physician.

**Conclusions:**

This study revealed the importance of digital media in the context of psoriasis, especially among women, individuals with shorter disease duration, and moderate mental disease severity. The lack of physicians’ digital media recommendations despite their patients’ desire to receive such and being more involved in health-related decisions seems to be a shortcoming within the physician-patient relationships. Physicians should guide their patients on digital media by recommending platforms with evidence-based information, thereby potentially creating an adequate framework for shared decision-making. Future research should focus on strategies to prevent the spread of false information on digital media and address the needs of patients and physicians to enhance health-related digital media offerings.

## Introduction

### Background

Psoriasis is a chronic inflammatory skin disease with a global prevalence ranging from 0.51% to 11.43% among adults [1]. Its etiology is multifactorial and influenced by genetic, environmental, and immunological factors [2]. Potential triggers include stress, infections, smoking, alcohol consumption, and specific medications like lithium [3]. The choice of psoriasis treatment depends on the disease severity, presence of psoriatic arthritis, comorbidities, and patient preferences. Established therapeutic options include topical therapy, phototherapy, oral-systemic therapy (eg, methotrexate), and biologics [3]. Individuals with psoriasis experience an increased mental burden due to stigmatization [4], a higher risk of addictive behavior [5], depression, and reduced overall well-being [6].

There has been a notable surge in the health-related use of digital media by individuals with dermatological conditions, particularly young and highly educated individuals facing socially burdensome skin conditions [7-9]. Digital media involves the transmission of information as digital data, encompassing different types of content such as audio, video, graphics, and text via digital devices such as computers, tablets, and smartphones. Examples range from social media platforms (eg, Facebook) and video streaming services (eg, YouTube) to digital radio stations and literary websites (eg, Wikipedia) [10]. In Germany, the internet has been increasingly used for accessing psoriasis-related information [11]. A prior study indicated that the psoriasis-related use of social media and the internet was more prevalent among individuals, who were young, had higher education, had higher impairment in skin disease-specific quality of life and severity, and those experiencing symptoms such as arthritis or involvement of facial or genital areas [12]. Additionally, Google and Facebook were found to be the most frequently used platforms among individuals affected by psoriasis [12].

Engaging with digital media platforms for health-related information provides several advantages. Notably, it empowers individuals by enhancing their knowledge, competence, and engagement in health decision-making processes [13]. Furthermore, it strengthens the physician-patient relationship and enables affected individuals to find answers, explore sensitive topics, and ask additional questions, from the comfort of their homes [14,15]. Blogs and digital support communities provide personalized insights and reflections and reduce feelings of loneliness and isolation [13].

However, there are also challenges regarding the use of health-related digital media. Regulating digital health information proves to be challenging due to the difficulties in maintaining quality control and the variability in the affected individual’s health information literacy [16]. Misleading or sensationalized content can result in harmful health decision-making, while the endorsement of unscientific practices further exacerbates the complexity [13,16]. Vulnerable individuals desiring empowerment may accept information without critical evaluation or may misinterpret it, thereby fostering erroneous knowledge and noncompliance [13,14]. Although the traditional role of physicians in providing personalized and evidence-based information may have been perceived as paternalistic, it ensured appropriate guidance based on individual comprehension [13].

Regarding the impact of digital media on the physician-patient relationship, a quantitative study indicated that physicians could recommend reputable digital media platforms; thereby, enhancing the quality of patient care [17]. Moreover, individuals with psoriasis tend to place greater faith in their physicians’ knowledge when disparities arise between information obtained from social media and the advice provided by physicians [12]. However, patients typically choose not to disclose their web-based research findings to their physicians [12].

Existing research predominantly centers on social media and the broader internet, primarily surveying patients within medical settings [7,12,18]. However comprehensive investigations that offer a more nuanced examination of digital media, encompassing individuals beyond the medical setting, remain conspicuously scarce [19].

### Objectives

The aims of this study were to (1) examine sociodemographic and medical factors influencing the health-related use of the internet among individuals affected by psoriasis, (2) rank specific digital media platforms and assess reasons for their use in the context of psoriasis, and (3) examine digital media within the physician-patient relationship from the perspective of individuals affected by psoriasis with and without dermatological treatment.

## Methods

### Study Design

For this cross-sectional study, an anonymous questionnaire was conducted among individuals affected by psoriasis in Germany between September 2021 and February 2022.

### Data Collection Methods

The questionnaire was designed by an interdisciplinary team, including 1 dermatologist, 2 epidemiologists, and 2 medical students. The questions are based on literature research [11,19,20] and qualitative interviews about digital media conducted by the same interdisciplinary team among psoriasis-affected individuals. The survey contained 20 items with multiple-choice and free-text answers and was split into 2 parts.

The first part contained the initial 12 items and could be filled out by every participant. This section covered demographic and medical information as well as information about psoriasis-related internet use: age (years)*,* sex*,* educational level, place of residence, disease duration (years), general disease severity (1 item question), mental burden of disease (1 item question), presence of psoriasis arthritis, dermatological treatment (yes or no), psoriasis knowledge, type of recruitment (digital media or clinical setting) and frequency of internet use regarding psoriasis. The items assessing the mental burden of disease, general disease severity, and psoriasis knowledge relied on self-reported data rather than validated instruments.

Participants who used the internet at least “less than once a month” for psoriasis-related information were considered internet users and eligible for the second part of the questionnaire. The second part contained the remaining 8 items of the questionnaire and asked about the following digital media platforms in the context of psoriasis: search engines, Wikipedia, general health websites, psoriasis-related websites, Facebook, other social media (Instagram, YouTube, and Twitter), and other forums or blogs. Additionally, the physician-patient relationship in the context of digital media was analyzed. For the second part, the following components and variables were used: frequency of each portal used, the best portal (single choice), search for treatment centers (yes or no), the communication of digital media use to the physician (yes or no), and if applicable physician response (positive, neutral, or negative), various statements regarding the physician-patient relationship, and an indication of reasons for using individual portals (multiple answer options). For the reasons for using individual portals, Facebook was considered separately since it is the most used social media platform in Germany and worldwide [21,22].

The survey was pilot-tested by 2 patients, resulting in minor changes in the wording of some questions. The final version of the questionnaire was incorporated into the platform REDCap (Research Electronic Data Capture; Vanderbilt University) to provide the digital questionnaire and to digitize the paper-based questionnaires.

### Sample Characteristics

Eligible for inclusion were individuals who were at least 18 years old, had psoriasis diagnosed by a physician (self-reported), and were able to answer a German questionnaire. Recruitment via digital media was initiated by sharing a link to the digital questionnaire in several psoriasis-related Facebook groups. These groups were general psoriasis support groups specifically aimed at German-speaking individuals. Collaboration with 2 prominent patient organizations (“Psoriasis-Netz” and “Farbenhaut”) was established, and these organizations shared the questionnaire link on their websites and in newsletters. The largest national psoriasis group in Germany (“Deutscher Psoriasis Bund eV”) also supported the recruitment efforts by disseminating the questionnaire link through their newsletter. Anyone who accessed the questionnaire via this link could participate in the survey. Moreover, patients were recruited personally at the Department for Dermatology and Allergy at the University Hospital of the Technical University of Munich. The clinical staff approached eligible inpatients consecutively, identified through their medical records as having psoriasis, and invited them to participate in the study. Similarly, clinical staff approached outpatients visiting the dermatology department for appointments consecutively. Both inpatients and outpatients were provided with detailed information about the study and, if they agreed to participate, were given a paper-based questionnaire to complete during their hospital stay or appointment. All participants, whether recruited via digital media or in person, received the same introductory information about the study to ensure consistency in understanding the study’s purpose and procedures.

### Ethical Considerations

The study was reviewed and approved by the local ethics committee of the Medical Faculty of the Technical University of Munich (reference 129/20 S). Informed consent was obtained from all participants. For the digital questionnaire, confirmation was necessary to complete the questionnaire. For the paper-based questionnaire, only those participants who confirmed the consent form were included in the statistical analysis. To preserve anonymity, the questionnaires did not ask for any identifying information such as name or address. For the paper-based questionnaires administered in the clinical setting, patients received the questionnaire from clinic staff who were not involved in this study. The patients then returned the filled-out questionnaires to the clinic staff who subsequently forwarded them to the study team. This process ensured that the questionnaires could not be traced back to individual participants. The participants in this study did not receive any form of compensation.

### Statistical Analysis

Data were descriptively analyzed. Absolute and relative frequencies were calculated for all categorical variables. For the reasons for using the individual portals*,* multiple answers were allowed so that the cumulative frequency or percentage could exceed 100%. For continuous variables, descriptive parameters (ie, mean and SD in case of normal distribution, median and IQR if not normally distributed, range) were determined, and histograms were constructed to evaluate distributions. The frequency at each portal was used and the variable frequency of internet use regarding psoriasis referred to the last 6 months and was categorized into “irregular use” (containing the answer options “not at all” and “less than once a month”) and “regular use” (“at least once a month,” “at least once a week,” and “daily”). The chi-square tests were used to analyze the association between the dichotomized variable frequency of internet use regarding psoriasis and the other nominal variables. In addition, Mann-Whitney and Kruskal-Wallis tests were used to assess the differences between the dichotomized variable frequency of internet use regarding psoriasis and the ordinal variables, as well as the nonnormally distributed metric variables disease duration and age. For the subsample of internet users, unpaired *t* tests were performed with the normally distributed metric variable age and the variable frequency of use of each portal to discover a possible difference in the age regarding the frequency of use of individual digital media platforms.

Univariate and multivariate binary logistic regressions were used to determine the associations between the dependent variable frequency of internet use regarding psoriasis and the following independent variables: age, disease duration, sex, education level, place of residence, general disease severity, mental burden of disease, presence of psoriasis arthritis, dermatological treatment, and psoriasis knowledge. To assess multicollinearity, Spearman correlation (*ρ*) and variance inflation factor (VIF) were used, with correlation *ρ*>0.7 or VIF≥10 being used cutoff values for multicollinearity [23,24]. If at least 1 cutoff value was exceeded, 1 of the affected variables had to be removed. Results of the regression analyses were reported with odds ratios (ORs), 95% CI, and *P* values.

For the frequency of each portal used, the variable best portal, and the indication of reasons for using individual portals, free-text responses were possible in addition to the answer options given. The free-text responses were recoded whenever they described a response option already listed.

Only the complete cases were used for all statistical analyses. The level of significance was set at α=.05 for all analyses. The SPSS statistics software (version 29; IBM Corp) was used for data analyses.

## Results

### Distribution of Total Data

**Figure 1:**
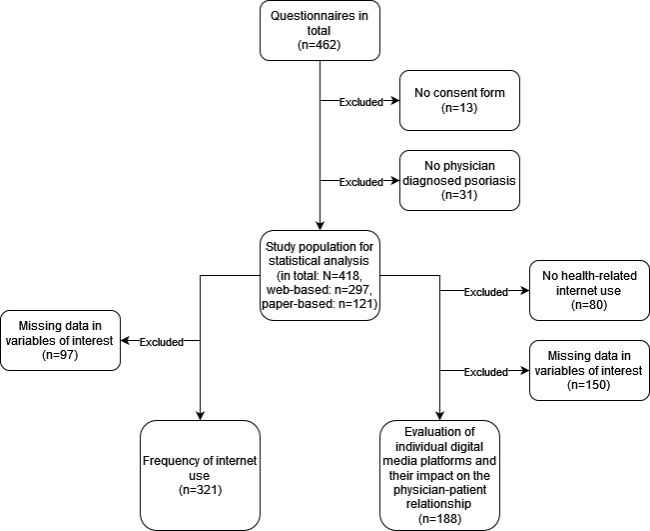
Flowchart of the study population and distribution.

In total, 462 individuals participated between September 2021 and February 2022. Initially, 44 participants who did not agree to the consent form (n=13) or whose psoriasis was not diagnosed by a physician (n=31) were excluded. Thus, based on these 418 questionnaires (digital: n=297 and paper-based: n=121), the missing data were excluded according to the research question (Figure 1).

### Frequency of Internet Use Regarding Psoriasis

In total, 321 participants were included with 195 (60.7%) being female (Table 1). The median age was 53.0 (IQR 41.0-61.0; range 18.0-82.0) years and the median disease duration was 22.0 (IQR 10.0-37.5) years with most of the participants reporting moderate or severe psoriasis (moderate: 147/321, 45.8%; severe: 142/321, 44.2%) and moderate to severe mental burden (moderate: 146/321, 45.5%; severe: 81/321, 29.3%). Overall, 71% of the participants (228/321) were treated by dermatologists. In the last 6 months, 54.2% (174/321) of participants used the internet regarding psoriasis regularly. Participants with a shorter disease duration were more likely to use the internet regularly for psoriasis-related information in the last 6 months (regular use: median 19.5, IQR 8-36 years vs irregular use: median 26, IQR 15-40 years; *P*=.007). There was no age-related difference in terms of the frequency of internet use regarding psoriasis (regular use: median 52.5, IQR 43-60.3 years vs irregular use: median 53, IQR 40-62 years; *P*=.81). In addition, the female sex and good or very good psoriasis knowledge were associated with a more regular internet use regarding psoriasis compared to male sex (*P*=.001) or less than good knowledge (*P*=.08). Individuals recruited from the clinical setting were less likely to use digital media regularly compared to individuals recruited through digital media (*P*<.001).

No violations of multicollinearity could be detected by checking the Spearman correlations and the VIF. The binary logistic regression analyses (Table 2) showed that participants with shorter disease duration (univariate: OR 0.99, 95% CI 0.97-1.00; multivariate: OR 0.98, 95% CI 0.96-1.00) and who were female (univariate: OR 2.13, 95% CI 1.35-3.36; multivariate: OR 1.70, 95% CI 1.03-2.81) compared to males were more likely to use the internet regarding psoriasis regularly. However, for age (univariate: OR 1.00, 95% CI 0.98-1.01; multivariate: OR 1.01, 95% CI 0.99-1.03), education (univariate: 0.78, 95% CI 0.50-1.21; multivariate: OR 0.88, 95% CI 0.54-1.44), and place of residence (univariate: OR 0.78, 95% CI 0.50-1.24; multivariate: OR 0.74, 95% CI 0.45-1.21), an association with the frequency of psoriasis-related internet use could not be found. Participants considering their psoriasis knowledge as good or very good had higher odds of regularly using the internet for psoriasis than people with less knowledge about their disease (multivariate: OR 1.97, 95% CI 1.01-3.83). In addition, the odds for regular psoriasis-related internet use were 80% higher in participants with a moderate mental burden of disease (multivariate: OR 1.80, 95% CI 1.01-3.20) compared to participants with no or mild mental distress.

**Table 1:** Characteristics of the study population overall (n=321) and stratified by the frequency of internet use regarding psoriasis.

Patients’ characteristics		Overall (n=321)	Internet use		*P* value
			Regular (n=174, 54.2 %)	Irregular (n=147, 45.8 %)	
**Age (years)**					.81^a^
	Median (IQR)	53.0 (41.0-61.0)	52.5 (43.0-60.3)	53.0 (40.0-62.0)	
	Range	18.0-82.0	21.0-82.0	18.0-81.0	
**Sex, n (%)**					.001^b^
	Male	126 (39.3)	54 (31)	72 (49)	
	Female	195 (60.7)	120 (69)	75 (51)	
	Diverse	0 (0)	0 (0)	0 (0)	
**Education level, n (%)**					.27^a^
	No access to university	144 (44.9)	83 (47.7)	61 (41.5)	
	Access to university	177 (55.1)	91 (52.3)	86 (58.5)	
**Place of residence, n (%)**					.30^a^
	Cities with up to 100,000 inhabitants	202 (62.9)	114 (65.5)	88 (59.9)	
	Cities with over 100,000 inhabitants	119 (37.1)	60 (35.5)	59 (40.1)	
**Disease duration (years)**					.007^a^
	Median (IQR)	22.0 (10.0-37.5)	19.5 (8.0-36.0)	26.0 (15.0-40.0)	
	Range	0.5-77.0	0.5-70.0	1.5-77.0	
**General disease severity, n (%)**					.09^c^
	Mild	32 (10)	14 (8)	18 (12.2)	
	Moderate	147 (45.8)	89 (51.1)	58 (39.5)	
	Severe	142 (44.2)	71 (40.8)	71 (48.3)	
**Mental burden of disease, n (%)**					.15^c^
	None or mild	94 (29.3)	43 (24.7)	51 (34.7)	
	Moderate	146 (45.5)	85 (48.9)	61 (41.5)	
	Severe	81 (25.2)	46 (26.4)	35 (23.8)	
**Presence of psoriasis arthritis, n (%)**					.26^b^
	Yes	166 (51.7)	95 (54.6)	71 (48.3)	
	No	155 (48.3)	79 (45.4)	76 (51.7)	
**Dermatological treatment by, n (%)**					.36^b^
	Yes	228 (71.0)	120 (69.0)	108 (73.5)	
	No	93 (29.0)	54 (31.0)	39 (26.5)	
**Psoriasis knowledge, n (%)**					.08^a^
	Less than good	55 (17.1)	24 (13.8)	31 (21.1)	
	Good or very good	266 (82.9)	150 (86.2)	116 (78.9)	
**Type of recruitment, n (%)**					<.001^b^
	Digital media	225 (70.1)	147 (84.5)	78 (53.1)	
	Clinical setting	96 (29.9)	27 (15.5)	69 (46.9)	

^a^Mann-Whitney test.

^b^Chi-square test.

^c^Kruskal-Wallis test.

**Table 2:** Results of the univariate and multivariate binary logistic regression with the frequency of internet use regarding psoriasis as a dependent variable and the several independent variables (n=321).

Variable		Univariate			Multivariate	
		OR^a^ (95% CI)	*P* value	OR (95% CI)		*P* value
Age		1.00 (0.98-1.01)	.86	1.01 (0.99-1.03)		.50
Duration of disease		0.99 (0.97-1.00)	.03	0.98 (0.96-1.00)		.008
Sex (reference: male)		2.13 (1.35-3.36)	.001	1.70 (1.03-2.81)		.04
Education (reference: No access to university)		0.78 (0.50-1.21)	.27	0.88 (0.54-1.44)		.62
Place of residence (reference: cities up to 100,000 inhabitants)		0.78 (0.50-1.24)	.30	0.74 (0.45-1.21)		.23
**General psoriasis severity (reference: mild)**						
	Mild to moderate	1.97 (0.91-4.27)	.09	1.62 (0.71-3.67)		.25
	Mild to severe	1.29 (0.59-2.78)	.52	0.96 (0.37-2.31)		.92
**Mental burden of disease (reference: none or mild)**						
	None or mild to moderate	1.65 (0.98-2.79)	.06	1.80 (1.01-3.20)		.046
	None or mild to severe	1.56 (0.86-2.84)	.15	1.96 (0.96-4.00)		.06
Presence of psoriasis arthritis (reference: no)		1.29 (0.83-2.00)	.26	1.11 (0.69-1.79)		.67
Dermatological treatment (reference: no)		0.80 (0.49-1.31)	.38	0.84 (0.50-1.44)		.53
Psoriasis knowledge (reference: less than good)		1.67 (0.93-3.00)	.09	1.97 (1.01-3.83)		.046

^a^OR: odds ratio.

### Evaluation of Individual Digital Media Platforms and Their Impact on the Physician-Patient Relationship

**Figure 2:**
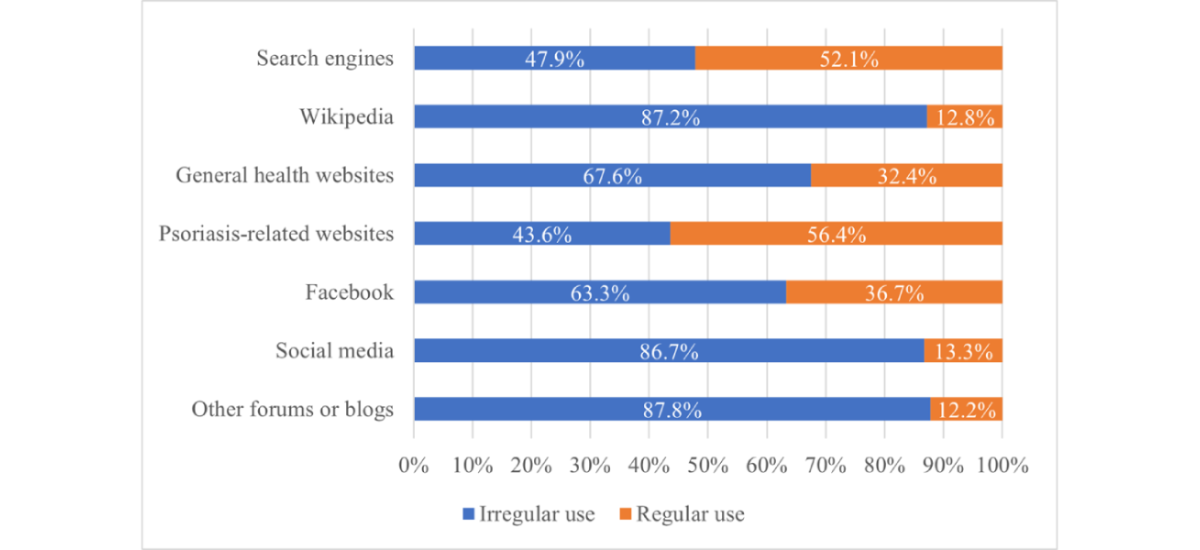
Several digital media platforms stratified by the frequency of use (irregular use: less than once a month, regular use: at least once a month; n=188).

In total, 188 participants who were considered internet users were included to evaluate digital media platforms and their impact on the physician-patient relationship. The mean age was 51.2 (SD 13.9, range 18.0-88.0) years. The most regularly used portals were psoriasis-related websites (106/188, 56.4%) and search engines (98/188, 52.1%; Figure 2).

The participants’ main reasons (Table 3) for using information websites and search engines were to seek information about psoriasis (132/188, 70.2%) and therapy options (133/188, 70.7%). Among the participants using social media and Facebook, the main reasons were to read experience reports from other patients (social media: 34/188, 18.1%; Facebook: 55/188, 29.3%), to seek information about the disease (social media: 32/188, 17%; Facebook: 36/188, 19.1%), and to engage with other affected people (social media: 27/188, 14.4%; Facebook: 58/188, 30.9%).

Regarding age, only Facebook showed a significant difference with people using Facebook regularly being generally younger (regular use: mean 47.4, SD 11.8 years vs irregular use: mean 53.4, SD 14.6 years; *P*=.004). By the majority of the participants (140/188, 74.5%) psoriasis-related websites were rated as the best digital portal regarding information about psoriasis.

**Figure 3:**
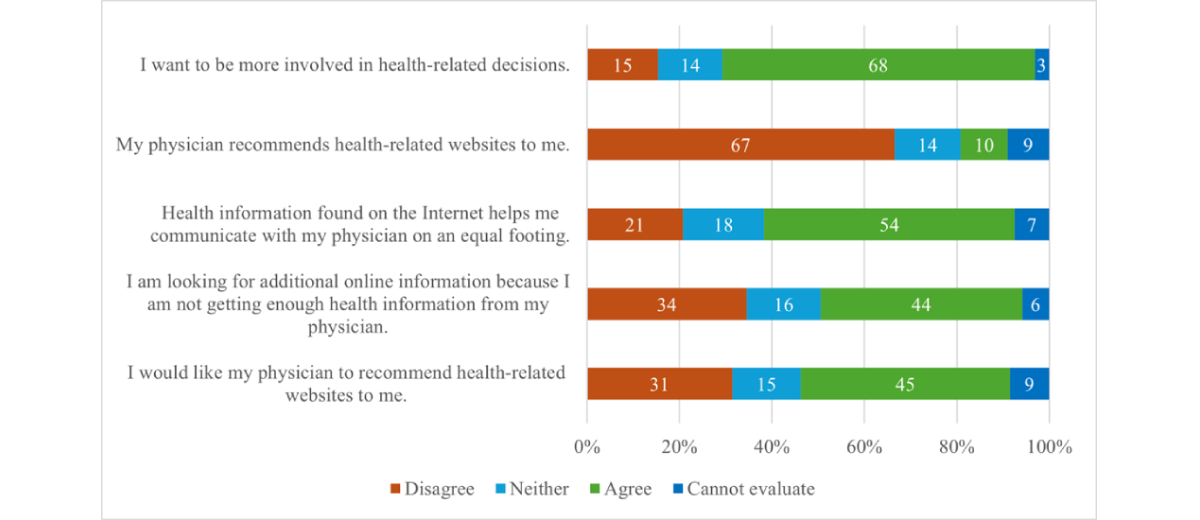
Statements about the physician-patient relationship (n=188) with the percentages of participants’ answers shown in the respective bars.

Overall, 72.3% (n=136) did web-based research on psoriasis treatment facilities with 43.1% (n=81) actually seeking consultation. In total, 75% (n=141) informed their physician about their web-based research and 77 (54.6%) received a neutral, 44 (31.2%) a positive, and 20 (14.2%) a negative reaction by their physician. Nearly 67.6% (n=127) wanted more involvement regarding health-related decisions. Approximately 44% (n=82) of the participants stated to search for additional information since the information provided by the physician was not sufficient. In addition, 66.5% (n=125) reported that their physicians have not recommended health-related websites, but 45.2% (n=85) wanted their physicians to do so. About 54.3% (n=102) stated that digital health-related information helped them to communicate with their physician on an equal footing (Figure 3).

**Table 3:** Reasons for using digital media from the participants’ perspective (n=188).

	Information websites and search engines	Social media	Facebook
I don’t use it, n (%)	10 (5.3)	139 (73.9)	112 (59.6)
For information about my disease, n (%)	132 (70.2)	32 (17)	36 (19.1)
For information about therapy options, n (%)	133 (70.7)	31 (16.5)	39 (20.7)
For information about alternative medicine, n (%)	83 (44.1)	21 (11.2)	30 (16)
To be able to speak more at the next physician´s appointment, n (%)	45 (23.9)	12 (6.4)	10 (5.3)
To read/check what my physician told me, n (%)	61 (32.4)	10 (5.3)	10 (5.3)
For information about the causes of my disease, n (%)	97 (51.6)	—^a^	—
To be able to have an exchange with other affected individuals, n (%)	—	27 (14.4)	58 (30.9)
To read experience reports from other affected individuals, n (%)	—	34 (18.1)	55 (29.3)

^a^Not available.

## Discussion

### Principal Results and Comparison With Prior Work

To gain a more differentiated understanding of the use of digital media and its influence on the physician-patient relationship to contribute to the appropriate care and support of those affected in the age of digital media, this study examined digital media use regarding psoriasis among people with and without dermatological treatment. Female sex, shorter disease duration, good self-assessed knowledge of psoriasis, and moderate mental burden were observed to be associated with regular psoriasis-related internet use. In addition, search engines and psoriasis-related websites were identified as the most frequently used platforms, especially for obtaining information about the disease and its therapy options. The fact that most physicians did not recommend health-related digital media to their patients despite their desire to receive such and being more involved in health-related decisions revealed shortcomings in the way physicians deal with digital media.

Our findings are in line with another study suggesting that women use the internet more for health-related information than men [25] although men generally used the internet more than women from 1997 until 2022 in Germany [26]. A prior study suggested that women perceived the internet as a more useful and entertaining medium for obtaining health information. Situational influences and a need to be well-informed contributed to women’s higher involvement in health-related internet searches [25].

Although no age differences between irregular and regular internet users were found in this work, it was reported that the elderly generally use the internet less than younger people [26]. The elderly may lack experience and comfort in seeking health information resulting in a lack of trust in the internet. Consequently, older adults not using the internet for health-related reasons but those having the desire to do so might profit from receiving training on identifying trustworthy digital media platforms that consistently provide accurate health information [27]. Furthermore, it is possible that a longer disease duration and therefore dealing with continuous struggles for a long time might lead to frustration among the affected individuals [28]. To avoid this emotion, emotion-oriented coping could lead to an avoidant behavior [29] and, therefore, probably to an avoidance of digital information seeking as well. In addition, individuals with a shorter disease duration may want to acquire more health-related information as they know less about their disease whereas individuals with a longer disease duration and therefore more experience and knowledge may use digital media irregularly, for example, to search for new findings.

The factors influencing psoriasis-based internet use in another study (eg, younger age and higher education) [12] do not or only slightly align with the results of our study. This could be explained by the study’s smaller sample size compared to the previous one (n=1520). Additionally, the previous study only included psoriasis-affected people from clinical settings.

One of the reasons for the limited use of social media platforms compared to, for example, psoriasis- or health-related websites, as identified in our study, may pertain to the quality of content present on certain platforms. For Facebook, it was priory revealed that the quality of psoriasis-related content was found to be inadequate, warranting improvements [19]. Another study found that approximately two-thirds of YouTube videos examined contained misleading or even dangerous content [30]. A further reason might be the age distribution in our study as a study from Germany found that the percentage of people using social media generally decreased as they got older [31]. In addition, some people may not want to discuss their disease on social media platforms because of the highly personal nature of the information. Concerns regarding the potential risks of sharing personal health data revolve around fears of discrimination by employers, insurance companies, and friends or family. These fears are intensified by stigmatized illnesses [32].

Search engines were one of the most consulted digital media when affected people sought health-related information [12,33]. Especially for these platforms a neutral controlling authority might be important to minimize false information. This could be achieved by ranking qualitative digital media platforms higher on search engines and thus making them more visible so that they are more likely to be accessed in health-related research on search engines. The quality of health-related digital media platforms could be ensured by including physicians in the development and supervision of these platforms [15]. Furthermore, there should be a regular exchange of opinions between physicians and patients to meet each other’s needs. In this study, search engines and information websites are mainly used when affected individuals want to inform themselves about the disease and therapy options. In comparison, a popular reason for the use of social media was to read experience reports from other affected people. However, especially when reading subjective reports from other affected individuals, it is crucial to control the spread of false information [19,30,34]. This could be achieved by a review process supervised by medical experts before these reports are visible to everyone.

To address the increasing desire to be involved in health-related decisions, it is necessary to reevaluate the conventional physician-patient relationship. It is crucial to adopt a medical approach that emphasizes the relationship and incorporates the patient’s view [35]. For patients seeking digital information, it was suggested to adopt a deliberative and participatory approach, which differs from the physician-centered and paternalistic models [36]. In this approach, physicians take on the role of educators and supporters, involving patients in a conversation to comprehend and choose from the various options presented during the decision-making process [37]. As identified in our study, the lack of digital media recommendations by physicians despite the patients’ desire to receive such should be a reminder for the physicians to promote and guide the digital information seeking of their patients [33,38]. Physicians should ensure that shared decision-making is based on reliable and evidence-based digital information. Nevertheless, the proportion of positive responses from physicians when patients inform them about their digital media use should increase to empower and guide patients who are interested in gaining more disease-related information. With this approach, patients can manage their disease more effectively and the physician-patient relationship may be improved by obtaining reliable digital health-related information [38-40]. However, it is important to acknowledge that physicians face a considerable burden with endorsing digital media, given the dynamic nature of content therein. Moreover, considering the breadth of diseases physicians treat, expecting them to be familiar with all available resources for each disease is demanding. This challenge could be mitigated by establishing a single high-quality, evidence-based platform that is supervised by medical professionals, thereby enabling physicians to recommend it.

### Strengths and Limitations

A strength of this study was the mixed study sample, achieved through recruitment inside the clinical setting but also from digital media platforms, thereby encompassing individuals who have limited contact with health care professionals or infrequent physician appointments. Furthermore, the questionnaire was tailored based on recently conducted qualitative interviews, ensuring the inclusion of target group-specific and current aspects.

Recall bias is likely when asking questions about digital media use in the past. Moreover, a selection bias must be assumed. The recruitment from a dermatology-specialized clinic likely resulted in the inclusion of participants with higher overall disease severity. Meanwhile, individuals recruited via digital media were more inclined to engage with digital media regarding their condition. The average age of the study population was rather high (median 53, IQR 41-61 years and mean 51.2, SD 13.9 years for the respective research questions) compared to the average age in Germany which was 44.7 years in 2021 [41]. However, the average age in Germany also included people under the age of 18 years. According to a German study, the older generation does not use the internet in general compared to the younger ones [26] whereby that study considered individuals aged 14 years and over. In addition, different categorizations of the variables may lead to different results, especially concerning regular and irregular digital media use. In addition, the sample size is small due to the exclusion of questionnaires with missing data, resulting in a considerable loss of information. Therefore, there may have been a higher variance in the estimators and a loss of statistical power. Moreover, the process of missing data itself may not be random, but rather systematic, leading to potential non-response bias in the results and therefore limiting the generalizability of the results. The participants' self-reported diagnosis of psoriasis was not confirmed through clinical evaluation which could potentially affect the validity of the results.

### Conclusions

The study underlines the significance of digital media in the health-related context, particularly with regard to women, individuals with shorter disease duration, and moderate mental disease severity. It also reveals the shortcomings within the physician-patient relationship that necessitate adaptation to the increasing digital media use. Physicians should guide their patients accordingly to ensure that they consume evidence-based information, thereby potentially creating an adequate framework for shared decision-making. Future studies should focus on analyzing and differentiating the individual digital media platforms in the context of psoriasis in more detail by assessing the quality of the information on the individual digital media platforms and additionally their potential for improving the physician-patient relationship. Additionally, to prevent the spread of false information on digital media, strategies against this prevalent problem should be developed in future studies. Furthermore, there should be a focus on the needs of psoriasis-affected people and physicians when discussing the future of health-related digital media. This will facilitate enhancements in, and, if necessary, expansion of the existing digital media offerings.

## Abbreviations

OR: odds ratio
REDCap: Research Electronic Data Capture
VIF: variance inflation factor

## Data Availability

The data sets generated or analyzed during this study are available from the corresponding author on reasonable request.
